# Development and pilot validation of a chemiluminescence immunoassay for monitoring immunologic changes during peanut oral immunotherapy using sIgE/sIgG4 ratio

**DOI:** 10.3389/falgy.2026.1850931

**Published:** 2026-07-01

**Authors:** Xiao Yang, Austin Vanderkallen, J. Joanna Yu, Jinguo Chen, Lili Wan, H. Henry Li, Zhongxin Li, Yuhuang Zhu, Iris Xi

**Affiliations:** 1 R&D Department, AdvanBio, Irvine, CA, United States; 2 R&D Department, Virant Diagnostics, Inc., Wheaton, MD, United States; 3 R&D Department, Institute for Asthma & Allergy, Chevy Chase, MD, United States; 4 AutoBio Diagnostics, Zhengzhou, China

**Keywords:** allergen specific IgG4, Ara h 2 and Ara h 6, Autolumo A2000 Plus, chemiluminescent immunoassay, longitudinal immunologic profiling, oral immunotherapy, peanut allergy, sIgE/sIgG4 ratio

## Abstract

**Background:**

The sIgE/sIgG4 ratio has been proposed as a dynamic measure of immune modulation during peanut oral immunotherapy (OIT). This study evaluated a chemiluminescent immunoassay for simultaneous quantification of peanut-specific sIgE and sIgG4 on the automated Autolumo A2000 Plus platform.

**Methods:**

Analytical performance was assessed for peanut, Ara h 2, and Ara h 6 sIgE and sIgG4. Method comparison with ImmunoCAP was performed across the measurable concentration range. In a separate retrospective pilot cohort of 23 peanut-allergic patients undergoing OIT, longitudinal sIgE, sIgG4 and sIgE/sIgG4 ratio trajectories were compared between A2000 and ImmunoCAP. Associations between ratio trajectories and OIT dose escalation were evaluated as an exploratory treatment-progression measure.

**Results:**

The A2000 Plus demonstrated high analytical sensitivity (sIgE LOQ < 0.05 kU/L; sIgG4 LOQ < 0.06 mg/L), precision (intra- and inter-assay CVs < 3% and <5%, respectively), and linearity (R² > 0.990). Method comparison with ImmunoCAP showed high positive percent agreement (82%–97%) and negative percent agreement (92%–100%), with strong concordance for log(sIgE) (*ρ* = 0.9041), and log(sIgG4) (*ρ* = 0.8464). Longitudinal analysis showed increased sIgG4 levels and decreased sIgE/sIgG4 ratio over time (*p* < 0.001), paralleling OIT dose escalation. Log(sIgE/sIgG4) ratio trajectories were strongly concordant between platforms across peanut, Ara h 2 and Ara h 6 (*ρ* = 0.900–0.917).

**Conclusion:**

The Autolumo A2000 Plus demonstrated strong analytical performance and captured expected immunologic trends during peanut OIT. These findings support further investigation of this platform as a potential complementary monitoring tool in OIT. Larger prospective studies are required to establish clinical utility.

## Introduction

Peanut allergy represents a leading cause of severe food-induced allergic reactions, affecting approximately 1% of children in industrialized countries, with a substantial proportion of cases continuing into adulthood (1, 2). The condition is mediated by allergen-specific IgE (sIgE), which drives mast cell and basophil activation and confers a significant risk of life-threatening anaphylaxis (3). Oral immunotherapy (OIT) is an emerging treatment that promotes desensitization through repeated allergen exposure. Several immunological parameters change during OIT and may serve as useful biomarkers for monitoring treatment progression and repsonse. These immunologic changes include increased allergen-specific IgG4 (sIgG4), decreased allergen-specific IgE (sIgE), and modulation of effector cell activation, each of which has been linked to the development of clinical tolerance (4–6).

Monitoring the response to OIT relies on a panel of complementary biomarkers, as no single definitive test exists. The oral food challenge (OFC) remains the clinical gold standard for assessing desensitization. Functional assays, such as the basophil activation test (BAT) which measures allergen-induced upregulation of activation markers (e.g., CD63, CD203c), provide evidence of reduced effector cell reactivity (7). Other markers, including allergen-specific IgA or cytokine responses from peripheral mononuclear cells (e.g., IL-10, IL-5, IL-13), offer valuable mechanistic insights, but are challenging to measure routinely due to a lack of standardized protocols (8, 9).

The sIgE/sIgG4 ratio is increasingly recognized as a valuable indicator of the immunological response to OIT. Mechanistically, elevated sIgG4 is thought to act as a blocking antibody, competing with sIgE for allergen binding and thereby inhibiting Fc*ε*RI-mediated degranulation (10, 11). Consequently, a persistently high sIgE/sIgG4 ratio is associated with ongoing clinical reactivity (12–15). While specific IgE to peanuts and its components Ara h 2 and Ara h 6 are valuable diagnostic parameters, sIgE levels alone often fail to distinguish between active allergy and clinical tolerance, as detectable sIgE can persist in the absence of clinical reactivity. Simultaneous measurement of sIgE and sIgG4 therefore provides a more comprehensive view of immune modulation during OIT. However, the sIgE/sIgG4 ratio is not currently recommended as a standalone diagnostic or prognostic biomarker, as clinically actionable cutoff values have not been established. Standardization of sIgG4 testing and validation of evidence-based cutoff values will be essential for the broader clinical adoption of this measure in monitoring immunologic changes during OIT.

The ImmunoCAP platform remains a widely used reference method for the *in vitro* quantification of sIgE in allergy diagnostics. Nevertheless, interference from cross-reactive carbohydrate determinants (CCDs) has been recognized as a potential source of false-positive results in ImmunoCAP-based assays (16, 17).. Enzyme-linked immunosorbent assays (ELISAs) is also a platform used to measure sIgG4 and sIgE, which can involve lengthy manual procedures, and limited automation. Accurate quantification of both sIgE and sIgG4 is important for calculating the ratio and evaluating its potential as a dynamic biomarker of immune modulation during OIT.

Chemiluminescence immunoassay (CLIA) provides a suitable technological platform for automated immunoglobulin measurement. CLIA detects antigen-antibody binding through light emission generated by a chemiluminescent reaction, producing a signal directly proportional to analyte concentration. Compared with conventional ELISA, CLIA can offer improved analytical sensitivity, broader dynamic range, reduced sample volume, faster assay processing, and compatibility with automated, high-throughput workflows (6, 18, 19). Non-cellulose-based CLIA assay formats may reduce matrix-associated CCD interference (20). These characteristics make CLIA a potentially useful approach for paired measurements of both sIgE and sIgG4. However, few validated CLIA platforms exist that can concurrently measure both peanut sIgE and sIgG4 with analytical performance comparable to the ImmunoCAP standard. This gap underscores the need for a robust assay system that combines technical excellence with practical clinical utility for OIT management (21).

The Autolumo A2000 Plus is an automated CLIA platform that enables simultaneous quantification of sIgE and sIgG4 from serum samples. In this retrospective pilot study, we evaluated a newly developed CLIA assay for concurrent measurement of sIgE and sIgG4 against peanut extract, Ara h 2 and Ara h 6 on the Autolumo A2000 plus analyzer. We first assessed the analytical performance of the sIgE and sIgG4 assays, followed by evaluation of analytical concordance with ImmunoCAP using peanut-, Ara h 2-, and Ara h 6-positive and -negative serum samples across the measurable concentration range. In addition, a separate set of stored longitudinal serum samples from 23 peanut-allergic patients undergoing OIT were analyzed to compare sIgE and sIgG4 ratio trajectories between platforms and to determine whether biomarker changes reflected OIT treatment progression. We hypothesized that the A2000 Plus platform would demonstrate strong analytical concordance with ImmunoCAP and reliably capture expected immunologic changes during peanut OIT.

## Materials and methods

### Sample collection

For method comparison between Autolumo A2000 Plus and ImmunoCAP platforms, de-identified serum samples were retrospectively obtained from a commercially available serum bank. Samples were selected to represent a clinically relevant range of allergen-specific antibody concentrations across the measurement range of both assays.

For real-world pilot investigation, we conducted a retrospective, longitudinal method-comparison study using stored serum samples from 23 patients with confirmed peanut allergy who were undergoing peanut OIT at a specialized allergy clinic. The study protocol was approved by the Salus IRB (IRB#24112). The cohort consisted of 19 males and 4 females and had a median age of 14 years (Supplementary Table S1). Each patient's samples were collected at three timepoints during OIT. Timepoint 1 baseline/early treatment (mea*n* ± SD: 3.4 ± 4.4 months; range: 0–15.5 months), Timepoint 2 mid-treatment (13.6 ± 2.1 months; range: 5.0–24.8 months), and Timepoint 3 late-treatment (22.2 ± 7.3 months; range: 8.6–34.0 months). A subset of patients at Timepoint 1 had already been receiving OIT for up to 15.5 months. All samples were de-identified, aliquoted, and stored at −80 °C until analysis.

### Analytical validation of sIgE and sIgG4 assays on Autolumo A2000 Plus

The performance of Analytical sensitivity, linearity and precision of the Autolumo A2000 Plus for peanut, Ara h2, and Ara h6 allergen-specific IgE and IgG4 assays were evaluated followingthe modified EP17-A2, EP06-A and EP05-A3 approaches, respectively.

Analytical sensitivity was evaluated by determining the Limit of Blank (LOB), Limit of Detection (LOD), and Limit of Quantitation (LOQ). The LOB was defined as the 95th percentile of measurements from blank samples. The LOD was calculated as the LOB + Cp × SD, where SD is the standard deviation of low-concentration samples, and Cp is the sample-size-adjusted multiplier corresponding to a 5% probability of false-negative results. The LOQ was established as the lowest concentration where the coefficient of variation (CV) was ≤ 20%.

Assay precision was evaluated by testing three concentration levels (low, medium, high) twice daily over two consecutive days. Intra- and inter-run CVs were calculated from this data.

Linearity was assessed by performing serial dilutions of high-titer clinical samples across the reportable range (sIgE: 0.1–100 kU/L; sIgG4; 0.1–30 mg/L). A linear regression model was used to determine the deviation from expected values.

For qualitative classification on the A2000 Plus platform, sIgE results ≥ 0.35 kU/L and sIgG4 results ≥ 0.3 mg/L were considered positive, using the corresponding ImmunoCAP reporting cutoffs as reference thresholds.

### Immunoglobulin quantification and cross-platform comparison

Allergen-specific IgE and IgG4 to whole peanut extract, Ara h 2, and Ara h 6 were quantified in parallel on the Autolumo A2000 Plus and the ImmunoCAP Phadia 250 systems (Thermo Fisher Scientific) using identical serum aliquots from the same thaw on the same day to eliminate freeze-thaw variability. Assay measurements were performed after no more than two freeze-thaw cycles. Operators were blinded to results from the comparator platform at the time of testing. Samples with results outside the measurement range on either platform were assigned the corresponding limit of measurement range for analysis.

The Autolumo A2000 Plus is a fully automated, high-throughput analyzer that employs magnetic microparticle-based CLIA technology. It offers a practical advantage for clinical monitoring through minimal sample volume requirements (20 µL per test) and rapid turnaround times (up to 200 tests per hour). All analyses on both platforms were performed using Manufacturer-validated reagents.

### Statistical analysis

PPA, NPA and total agreement of Autolumo A2000 Plus were calculated relative to ImmunoCAP as the reference method. 95% confidence intervals (CIs) were computed using the modified Wald method. The number of paired samples per analyte ranged from 87 to 126 for sIgE and 63–79 for sIgG4, reflecting differences in sample availability within the archive.

For each allergen (peanut, Ara h 2, and Ara h 6), sIgE/sIgG4 ratios were log_10_ transformed prior to analysis. Longitudinal changes in sIgE, sIgG4, and their ratios over the OIT course were analyzed using mixed-effects models with Geisser-Greenhouse correction, followed by Tukey's *post hoc* test applied for pairwise comparisons.

Spearman rank correlation coefficients were computed for all paired log-transformed sIgE, sIgG4 and sIgE/sIgG4 ratios, and visualized with linear regression plots. Agreement between Autolumo A2000 Plus and ImmunoCAP was further assessed by Bland-Altman analysis for sIgE and sIgG4. The difference between A2000 Plus and ImmunoCAP measurements was plotted against the mean of two measurements for each paired sample. All analyses were performed using GraphPad Prism version 10.0 (GraphPad Software, Inc.).

### Bias mitigation

Several measures were implemented to minimize potential bias. All serum samples were de-identified prior to analysis. Data collection, laboratory testing, statistical analysis, and interpretation of findings were performed exclusively by authors independent of AutoBio Diagnostics, as documented in the Author Contributions section. The retrospective study design and use of pre-collected stored samples precluded prospective manipulation of sample selection or collection protocols.

## Results

### Analytical performance of sIgE and sIgG4 assays on Autolumo A2000 Plus platform

**Figure 1:**
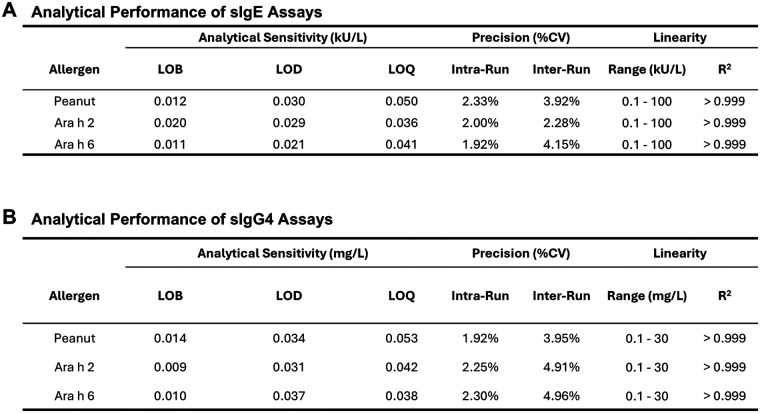
Analytical performance of sIgE and sIgG4 assays. Analytical performance of the allergen-specific **(A)** IgE and **(B)** IgG4 assays on the Autolumo A2000 Plus system, including sensitivity [limit of blank [LOB], limit of detection [LOD], and limit of quantitation [LOQ]], precision [intra- and inter-run coefficient of variation (%CV)], and linearity (R² and linear range). All assays demonstrated high sensitivity, reproducibility, and linearity across clinically relevant concentrations.

The Autolumo A2000 Plus platform demonstrated high analytical sensitivity across all assays. For sIgE, the limits of blank (LOB), detection (LOD), and quantitation (LOQ) ranged from 0.011 to 0.020 kU/L, 0.021–0.030 kU/L, and 0.036–0.050 kU/L, respectively. For sIgG4, the LOB, LOD, and LOQ ranged 0.009–0.014 mg/L, 0.031–0.037 mg/L, and 0.038–0.053 mg/L, respectively (Figures 1A,B).

The assay also showed excellent precision, with intra-run coefficients of variation (CVs) of 1.92%–2.33% for sIgE and 1.92%–2.30% for sIgG4, and inter-run CVs ranged of 2.28%–4.15% for sIgE and 3.95%–4.96% for sIgG4 (Figures 1A,B).

Linearity was confirmed for all assays, with coefficients of determination (R²) exceeding 0.999 (Figures 1A,B). These results support accurate quantitation across the reportable range of the assays.

### Method comparison of the Autolumo A2000 Plus and ImmunoCAP

**Figure 2:**
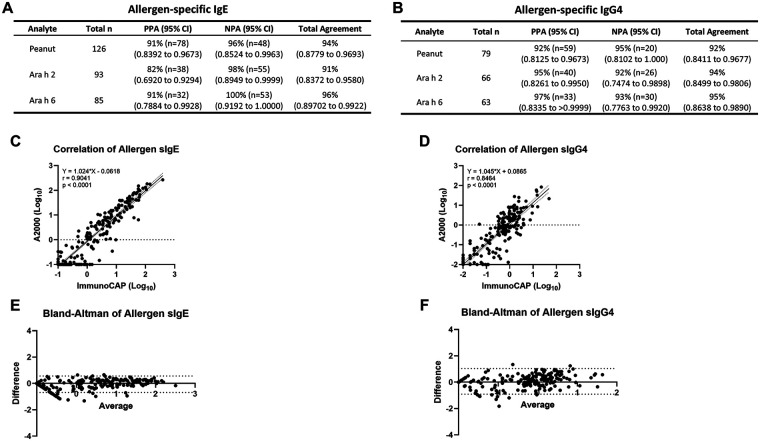
Analytical comparison between A2000 Plus and ImmunoCAP. **(A,D)** The Autolumo A2000 Plus demonstrated high qualitative agreement with ImmunoCAP for sIgE and sIgG4 measurements of peanut, Ara h 2, and Ara h 6, as assessed by sensitivity, specificity, and total class agreement compared to ImmunoCAP. **(B,E)** sIgE and sIgG4 measurement for peanut, Ara h 2, and Ara h 6 together showed strong correlation between the Autolumo A2000 Plus and ImmunoCAP platforms through Spearman's rank correlation. The correlation coefficients (ρ) for sIgE and sIgG4 were 0.9041 and 0.8464, respectively. **(C,F)** Bland-Altman diagrams plot differences (ordinate values, in log_10_) in sIgE and sIgG4 measurements between A2000 and ImmunoCAP against average values (abscissa, log_10_). R, Spearman's ρ**.**

The Autolumo A2000 Plus demonstrated high qualitative agreement with ImmunoCAP for all six peanut-related analytes. For sIgE assays (Figure 2A), positive percent agreement (PPA) ranged from 82% for Ara h 2 (95% CI, 0.69–0.93), 91% for Ara h6 (95% CI, 0.79–1.00) to 91% for peanut extract (95% CI, 0.84–0.97), while negative percent agreement (NPA) ranged from 98% for Ara h2 (95% CI, 0.89–1.00), 100% for Ara h 6 (95% CI, 0.92–1.00), to 96% for peanut extract (95% CI, 0.85–1.00); *n* = 85–126 per analyte). Total agreement for sIgE was ≥90% across three analytes, with 94% for peanut extract (95% CI, 0.88–0.97; *n* = 126), 91% for Ara h 2 (95% CI, 0.84–0.96; *n* = 93), and 96% for Ara h 6 (95% CI, 0.90–0.99; *n* = 85).

For sIgG4 assays (Figure 2B), positive percent agreement (PPA) ranged from 95% for Ara h 2 (95% CI, 0.83–1.00), 97% for Ara h6 (95% CI, 0.83–1.00) to 92% for peanut extract (95% CI, 0.81–0.97), while negative percent agreement (NPA) ranged from 92% for Ara h2 (95% CI, 0.75–0.99), 93% for Ara h 6 (95% CI, 0.78–0.99), to 95% for peanut extract (95% CI, 0.81–1.00); *n* = 85–126 per analyte). Total agreement for sIgE was ≥90% across three analytes, with 92% for peanut extract (95% CI, 0.84–0.97; *n* = 79), 94% for Ara h 2 (95% CI, 0.85–0.98; *n* = 66), and 95% for Ara h 6 (95% CI, 0.86–0.99; *n* = 63).

Quantitative agreement between A2000 and ImmunoCAP platforms was strong for both sIgE and sIgG4. Spearman correlation analysis demonstrated strong rank-order agreement across the measurement range for sIgE (*ρ* = 0.9041, *n* = 310; Figure 2C) and sIgG4 (*ρ* = 0.8464, *n* = 208; Figure 2D). Bland–Altman analysis showed minimal mean bias for sIgE measurements (mean bias, −0.06435; 95% limits of agreement, −0.6930–0.5643; Figure 2E) and sIgG4 measurements (mean bias, 0.06316; 95% limits of agreement, −0.9071–1.033; Figure 2F), supporting a good analytical concordance between the two platforms.

### Longitudinal changes in peanut-specific IgE and IgG4 during oral immunotherapy

**Figure 3:**
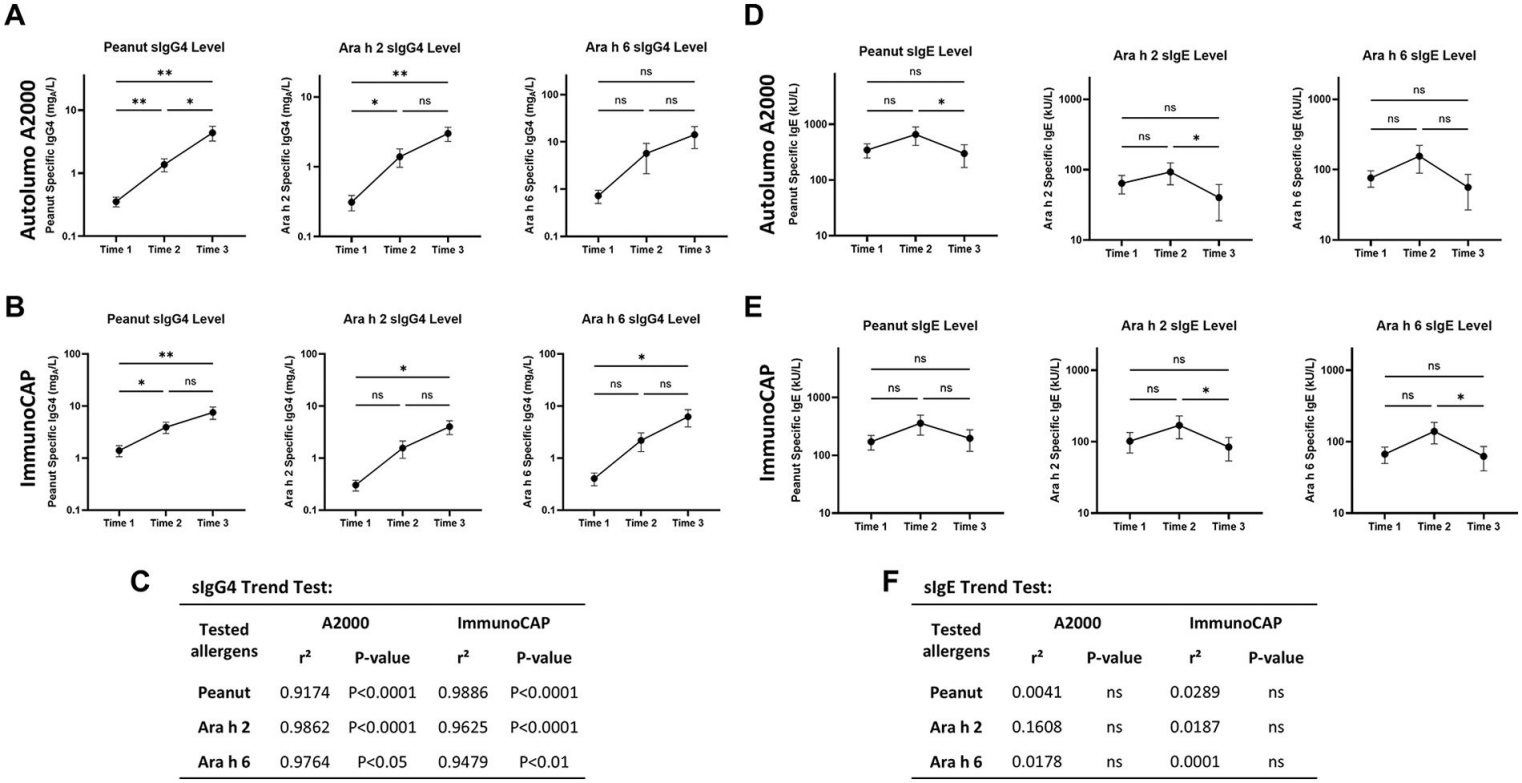
Longitudinal changes in peanut-specific antibody levels during OIT. **(A,B)** Levels of sIgG4 to peanut, Ara h 2, and Ara h 6 increased significantly over time, as measured on both the Autolumo A2000 Plus and ImmunoCAP platforms. **(C)** Linear trend analysis confirmed significant positive slopes for sIgG4. **(D,E)** In contrast, peanut-specific IgE levels showed no significant decline throughout treatment. **(F)** This was confirmed by trend analysis which indicated no significant downward slope for sIgE. *P < 0.05, **P < 0.01, ns, not significant.

Analysis of longitudinal serum samples (*n* = 23) revealed a statistically significant, time-dependent increase in peanut-specific IgG4 levels as quantified by both the Autolumo A2000 Plus and ImmunoCAP platforms (*P* < 0.0001; Figures 3A,B). A similar significant increase was observed for Ara h 2 and Ara h 6 sIgG4, with peak concentrations observed at the final timepoint. Trend analysis confirmed a significant positive linear trend for sIgG4 across all allergens (Figure 3C).

In contrast, peanut-specific IgE levels showed no significant decline at the intermediate timepoint (Timepoint 2) on either platform, the modest decrease observed by Timepoint 3 was not statistically significant (*P* > 0.05; Figures 3D,E). A similar pattern was observed for Ara h 2 and Ara h 6 sIgE, which showed variable reductions among patients without a consistent downward trend. Consequently, overall trend analysis confirmed the absence of a significant linear decrease in sIgE for any allergen (*P* > 0.05; Figure 3F).

### Significant reduction in sIgE/sIgG4 ratio over the course of OIT

**Figure 4:**
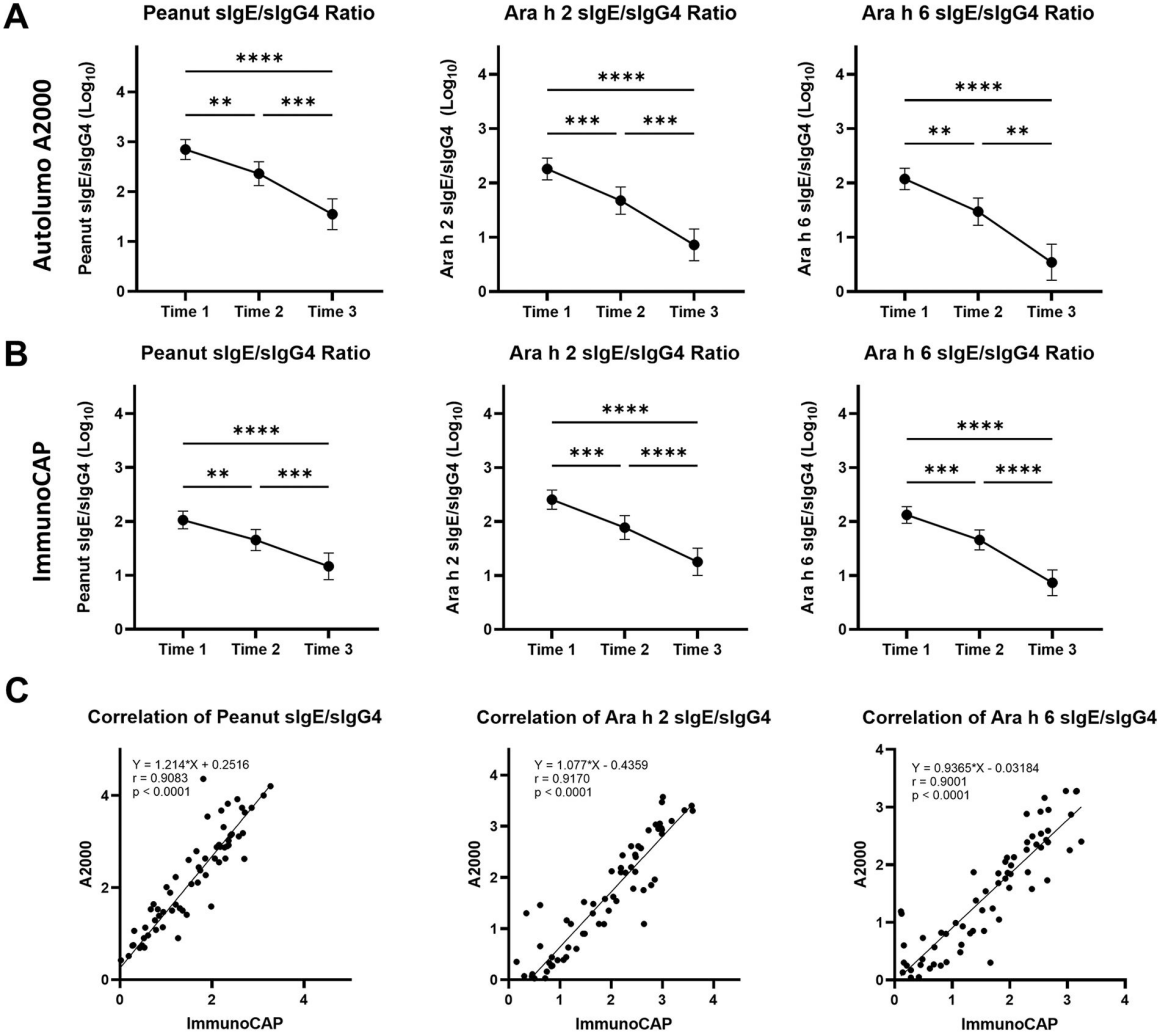
Time-dependent decline in the sIgE/sIgG4 ratio during peanut OIT. Log₁₀-transformed sIgE/sIgG4 ratios for peanut, Ara h2, and Ara h6 are shown for the **(A)** Autolumo A2000 Plus and **(B)** ImmunoCAP platforms. A significant time-dependent decrease in the sIgE/sIgG4 ratio was observed for all allergens on both platforms. **(C)** Scatter plots display the Spearman correlation of sIgE/sIgG4 ratios for peanut, Ara h 2, and Ara h 6 between the A2000 and ImmunoCAP platforms. Correlation coefficients (*ρ*) were 0.9083 for peanut, 0.9170 for Ara h 2, and 0.9001 for Ara h 6, indicating high concordance between the two systems. **P < 0.01, ***P < 0.001, **** *P* < 0.0001.

Analysis of the log-transformed sIgE/sIgG4 ratio revealed a significant time-dependent decrease for peanut, Ara h 2, and Ara h 6 on both the ImmunoCAP and Autolumo A2000 Plus platform (*P* < 0.0001; Figures 4A,B). The greatest reduction occurred between Timepoint 1 and 3, with a decrease of more than one log unit in the majority of patients. Trend analysis confirmed a significant linear downward trajectory for all three allergens on both platforms (Supplementary Table S2). This reduction in sIgE/sIgG4 ratio occurred in parallel with a significant time-dependent increase in peanut OIT dosage treatment (Supplementary Figure S1), supporting an association between serologic changes and OIT treatment progression. Together, these findings are consistent with prior reports that OIT is characterized by increase in sIgG4, variable changes in sIgE, and a declining sIgE/sIgG4 ratio as a dynamic marker of immunologic modulation (10).

### Correlation between Autolumo A2000 Plus and ImmunoCAP platform

Spearman correlation analysis revealed strong agreement between the Autolumo Plus and ImmunoCAP platforms for log-transformed sIgE/sIgG4 ratios across all allergens: peanut (*ρ* = 0.9083), Ara h 2 (*ρ* = 0.9170), and Ara h 6 (*ρ* = 0.9001; all *P* < 0.0001 (Figure 4C). These results align with previous studies validating A2000 plus platforms against ImmunoCAP for allergen-specific antibody detection, particularly where Spearman correlation is used due to non-normality in immunoglobulin distributions (20, 22).

## Discussion

The Autolumo A2000 Plus CLIA system demonstrated strong analytical performance across all evaluated parameters, including high analytical sensitivity for peanut extract, Ara h 2, and Ara h 6, excellent linearity (R² > 0.99), and high precision (intra- and inter-assay CVs < 3% and 5%, respectively). These characteristics support reliable quantification of immunologic changes over the concentration range encountered during OIT and provide the analytical foundation for further investigation of this platform as a monitoring tool in this setting. Inter-platform analytical concordance with ImmunoCAP was strong (Spearman's *ρ* = 0.9041 for sIgE and 0.8464 for sIgG4), consistent with published validations of chemiluminescence immunoassays for allergen-specific antibodies in which non-parametric concordance metrics are appropriate given the non-normal distribution of immunoglobulin concentrations in clinical populations (20, 22–24). Key technical and workflow characteristics of the ImmunoCAP and A2000 Plus platforms are summarized in Supplementary Table S3. This comparison is intended to contextualize assay format and operational consideration, rather than to demonstrate clinical, economic, or workflow superiority.

Longitudinal profiling with the A2000 Plus revealed significant immunologic shifts. We observed marked increases in sIgG4 across all allergen targets. In contrast, sIgE levels did not show a significant decline, aligning with previous reports that sIgE can remain elevated despite clinical improvement, especially in early-phase OIT (25, 26). Consequently, the sIgE/sIgG4 ratio demonstrated a consistent and marked reduction over time. This finding aligns with prior studies identifying the sIgE/sIgG4 ratio as a composite biomarker reflecting a shift toward a tolerogenic immune profile (27–32). The ability of the A2000 Plus platform to detect these expected shifts confirms its sensitivity for monitoring therapeutic responses and corroborates the ratio's role as a potential serological marker (10).

While these observations support the ratio's potential as a dynamic biomarker for monitoring the immunological trajectory during peanut OIT, it is important to note that current allergy practice guidelines have not endorsed the use of sIgE/sIgG4 ratio as a standalone diagnostic or prognostic tool (33, 34). Instead, sIgE/sIgG4 measurement may serve as complementary tools that provide complementary mechanistic insight when interpreted alongside clinical history and, in future studies, standardized clinical endpoints such as OFC outcomes. Its role may be particularly valuable for monitoring immune changes during OIT and informing future studies of individualized monitoring strategies, though further longitudinal studies are needed to establish clinical thresholds and validate their predictive utility across diverse patient populations.

Interpretation of longitudinal sIgE and sIgG4 changes during peanut OIT should account for substantial biological and study-design variability. Prior studies have shown that peanut OIT typically induces increases in peanut sIgG4 while peanut sIgE may rise early during treatment and decline later, indicating that sIgE trajectories are time-dependent and may not follow a uniform pattern across patients (35, 36). Inter-individual variability in antibody responses is also expected, as OIT responsiveness differs across patients and current immunologic markers remain imperfect surrogates for clinical outcome (35). Patient age may further contribute to heterogeneity, because younger patients appear to have a greater likelihood of successful desensitization or remission in some cohorts, although recent analyses suggest that baseline peanut sIgE may be a stronger predictor than age alone (37–39). In the present study, additional variability was introduced by heterogeneous OIT duration at the first sampling timepoint and variable intervals between serial sample collections, which may influence the apparent magnitude and direction of biomarker change. Therefore, observed change in sIgE/sIgG4 ratio should be interpreted as exploratory immune-response patterns rather than definitive predictors of clinical benefit.

The Autolumo A2000 Plus is, to our knowledge, the first automated CLIA-based platform reported for concurrent measurement of sIgE and sIgG4 in a single testing workflow. In this pilot study, the platform demonstrated strong analytical performance and concordance with ImmunoCAP for peanut-related sIgE and sIgG4 measurements. Its ability to quantify paired antibody isotypes from the same serum sample may support longitudinal assessment of immunologic changes during OIT. However, because OFC outcomes, sustained unresponsiveness, formal clinical validation were not performed, broader clinical implementation will require further prospective evaluation.

### Limitations

Despite the strengths, several limitations of this pilot study must be acknowledged. First, the sample size was small (*n* = 23), limiting statistical power for clinical inference and the precluding multivariable modeling of covariates such as age, sex, and treatment duration. All longitudinal analyses are exploratory and require confirmation in adequately powered prospective cohorts. Second, the retrospective design introduced heterogeneity in sampling windows and treatment duration. Timepoint 1 spanned a wide range (0–15.5 months; mean 3.4 ± 4.4 months), meaning that some patients were not treatment-naïve at first sampling. All longitudinal comparisons therefore reflect changes from each patient's first available timepoint rather than from a true pre-treatment baseline, and causal interpretation of within-subject trajectories should be made with caution. Third, clinical outcome data, including symptom scores and sustained unresponsiveness, were unavailable in this retrospective cohort. The observed serologic changes, even though shown the trend of changes with treatment course, should not be interpreted as validated endpoints for treatment decisions. A large cohort of clinical validation with comprehensive design is needed to further evaluate this marker's clinical utility. Fourth, all samples were collected and analyzed within a single specialized center. Inter-laboratory reproducibility of the newly developed sIgE and sIgG4 assays has not been assessed, and multicenter validation is required before the platform can be considered broadly applicable. Future prospective studies incorporating standardized clinical endpoints, OFC outcomes, appropriate control groups, age stratification, and multicenter design will be essential to establish the clinical significance of the sIgE/sIgG4 ratio measured on the A2000 platform.

## Conclusion

In summary, this single-center pilot investigation introduced and analytically evaluated a CLIA-based assay for concurrent quantification of peanut, Ara h 2, and Ara h 6 sIgE and sIgG4 on the Autolumo A2000 Plus platform. The assay demonstrated strong analytical sensitivity, precision, linearity, and concordance with ImmunoCAP, supporting its technical feasibility for paired sIgE/sIgG4 measurement. Longitudinal analysis further showed that declining sIgE/sIgG4 ratios paralleled OIT dose escalation, supporting the potential use of this platform for exploratory immunologic monitoring during peanut OIT. These findings establish an analytical foundation for larger prospective studies with standardized sampling, clinical endpoints, and treatment-response measures to determine the clinical significance sIgE/sIgG4 ratio trajectories and the potential role of this platform in OIT management.

## Data Availability

The original contributions presented in the study are included in the article/Supplementary Material, further inquiries can be directed to the corresponding author.

## Supplementary material

The Supplementary Materialfor this article can be found online at: https://www.frontiersin.org/articles/10.3389/falgy.2026.1850931/full#supplementary-material

Supplementary Table S1 Patient Demographics and Clinical Characteristics. Demographic and clinical characteristics of the 23 patients undergoing peanut oral immunotherapy (OIT). Serum samples were collected at three treatment timepoints and analyzed for specific IgE (sIgE) and specific IgG4 (sIgG4) to peanut, Ara h2, and Ara h6.

Supplementary Table S2 sIgE/sIgG4 Ratio Trend Test. A time-dependent decrease in the sIgE/sIgG4 ratio was observed for peanut, Ara h 2, and Ara h 6 across both platforms. Both platforms detected a consistent and significant downward trend across all allergens.

Supplementary Table S3 Comparison of Key Technical and Workflow Characteristics. Operational comparisons are provided for context only, as formal cost-effectiveness, personnel-time, turnaround time, and workflow impact analyses were not performed.

Supplementary Figure S1 Time-dependent Increase in Peanut OIT Dosage. Peanut OIT treatment dose increased significantly across the three timepoints in the retrospective pilot cohort, consistent with treatment progression during OIT. ***P* < 0.01, ****P* < 0.001, ns, not significant.
