# Supplementary material for: Development and pilot validation of a chemiluminescence immunoassay for monitoring immunologic changes during peanut oral immunotherapy using sIgE/sIgG4 ratio
Source: Front Allergy. 2026 Jul 1;7:1850931. doi: 10.3389/falgy.2026.1850931 (PMC13368872; doi:10.3389/falgy.2026.1850931)
Supplement: Supplementary file 5 [file Supplementaryfile4.pdf]

Supplementary Materials

Supplementary Table 1. Patient Demographics and Clinical Characteristics. Demographic and clinical characteristics of the 23 patients undergoing peanut oral immunotherapy (OIT). Serum samples were collected at three treatment timepoints and analyzed for specific IgE (sIgE) and specific IgG4 (sIgG4) to peanut, Ara h2, and Ara h6.

Supplementary Table 2. sIgE/sIgG4 Ratio Trend Test

A time-dependent decrease in the sIgE/sIgG4 ratio was observed for peanut, Ara h 2, and Ara h 6 across both platforms. Both platforms detected a consistent and significant downward trend across all allergens.

Supplementary Figure 1. Time-dependent Increase in Peanut OIT Dosage

Peanut OIT treatment dose increased significantly across the three timepoints in the retrospective pilot cohort, consistent with treatment progression during OIT. **$P < 0.01$, ***$P < 0.001$, ns, not significant.

Supplementary Table 3. Comparison of Key Technical and Workflow Characteristics

Operational comparisons are provided for context only, as formal cost-effectiveness, personnel-time, turnaround time, and workflow impact analyses were not performed.
